# Challenges in the Implementation of Measurement Feedback Systems

**DOI:** 10.1007/s10488-015-0697-y

**Published:** 2015-10-30

**Authors:** Kim de Jong

**Affiliations:** Department of Clinical Psychology, Institute of Psychology, Leiden University, Wassenaarseweg 52, 2333 AK Leiden, The Netherlands

**Keywords:** Outcome monitoring, Feedback, Implementation

## Abstract

This commentary on the articles published in the special section on the development and implementation of measurement feedback systems (MFSs) discusses three challenging themes in the process of MFS implementation: design and planning, organizational context, and sustainability and unintended consequences. It is argued that the implementation of MFSs is complex, but is an important step in improving outcomes in routine care for children and young persons.

In the past decade, the implementation of measurement feedback systems (MFSs), also referred to as progress feedback or (routine) outcome monitoring, has taken a leap worldwide. Several countries have mandated the use of MFSs as part of routine care (e.g. United Kingdom, Australia, Norway, the Netherlands), and in other countries large public or private initiatives exist (e.g. United States, Germany, Chile). By using an MFS, the client’s progress in treatment is tracked by frequent administration of standardized measures. The MFS supports the clinician in deciding to adapt treatment when insufficient progress has been made. There are several feedback systems available (e.g. OQ Measures, PCOMS, TOP), and by now MFSs have been introduced in a variety of settings (inpatient, outpatient, group and individual therapy), populations (e.g. youth, adults, elderly) and disorders (e.g. addiction, common mental disorders, eating disorders) (Bickman et al. 2011; Crits-Christoph et al. 2010; Kraus et al. 2005; Lambert et al. 2004; Miller et al. 2005; Probst et al. 2013; Simon et al. 2013). Some studies have found large effects of using MFSs (Shimokawa et al. 2010), but a recent review suggests that the effects can vary substantially over studies (Krägeloh et al. 2015). A potential explanation for this variation in effectiveness might be the way in which MFSs have been implemented (de Jong 2014). For example, two recent studies found that half of the clinicians did not use the feedback they were provided with (e.g. De Jong et al. 2012; Simon et al. 2012). As such, it is worth taking a closer look at the processes associated with the implementation of MFSs, as the articles in this special section aim to do. Dixon-Woods et al. (2012) analyzed evaluation reports from a large number of quality improvement programs in the UK, and identified three themes for implementation: (1) Design and planning of the improvement intervention; (2) Organizational and institutional contexts, professions and leadership; and (3) Beyond the intervention: sustainability, spread and unintended consequences. The articles in this special section will be discussed within the context of these themes.

## Design and Planning

The first implementation theme is the design and planning of the introduction of the MFS. The way Dixon-Woods et al. (2012) define this, it includes the process of convincing clinicians, staff and management that there is a problem (e.g. outcomes are not good enough), for which the implementation of the MFS is the solution (e.g. MFSs improve outcomes). Although none of the articles in this section mention explicitly how they have addressed this issue, in Gleacher et al. (2015) a clinician mentions that once (s)he saw the value of the MFS, (s)he really started to buy into it. Research has shown that clinicians can have quite negative attitudes towards MFSs (Walter et al. 1998), and that they predict active use of the MFS (De Jong et al. 2012). That makes them an important target in the implementation process.

The design of the MFS is another important factor within this theme. MFS design has been discussed in detail in several of the articles in this section (Bruns et al. 2015; Nadeem et al. 2015; Steinfeld et al. 2015). Given the technical complexity of MFSs, and a group of users that is not necessarily “computer savvy”, the design of the MFS is extremely important. Bickman and colleagues report that problems with their MFS was mentioned by clinicians as the main barrier for implementation in their study (Bickman et al. 2014). MFS needs to fit their users’ needs. Specifically regarding the technology aspects Lyon et al. (2015) provide a framework for optimizing existing software packages to fit the needs of mental health care organizations, in which they put a strong emphasis on the involvement of future users. Given that the technical aspect is one of the complicating factors of implementing a MFS, compared to other implementation processes, the article by Lyon et al. is a valuable addition to the implementation literature.

## Organizational Context

The second theme that Dixon-Woods and colleagues address is organizational and institutional contexts, professions, and leadership. This is an important theme in the articles in this special section. Both Nadeem et al. and Gleacher et al. conclude that higher organizational and leadership support improved implementation of the MFS. Interviews with clinicians in the article by Bickman et al. shed a light on what type of support was particularly helpful: on-site implementation support, and having a local champion in using the MFS. These results are in line with the conclusion of Aarons et al. (2014) that leaders have a key role in promoting an implementation climate. Aarons et al. stress that alignment across multiple levels of leadership is especially important. A study by Torrey et al. (2012) found that implementation success was correlated with leadership devoted to redesigning the work flow and reinforcing implementation though measurement and feedback. Dixon-Woods et al. (2012) suggest that a ‘quieter’ leadership style might be more succesfull: fewer bombastic declarations and more working to facilitate collaboration.

## Sustainability and Unintended Consequences

The third theme mentioned by Dixon-Woods et al. (2012) refers to sustainability and unintended consequences. With the exception of Steinfeld et al. in the studies in this special issue the implementation of the MFSs was heavily supported by research teams. This may make sustainability a potential issue. Especially when MFSs were implemented as part of a specific project, there appears to be a risk that clinicians and managers lose interest at the project’s end, when they are faced with new, competing priorities (Dixon-Woods et al. 2012). As Lyon et al. point out, the adaptation of the MFS is an ongoing process, one that in my experience is often underestimated at the start of the process by management, researchers and even the software engineers. However, it is possible to implement a MFS successfully without external support. For example, Steinfeld et al. (this issue) obtained impressive percentages of complete data, and a majority of clinicians accessing the feedback before seeing the patient without external support. The key to their success seems to be keeping the process simple and taking one small step at a time.

The issue of unintended consequences is a complex one. Research on the efficacy of MFSs has predominantly taken place in adult populations. To my knowledge, in youth mental health care only two randomized controlled studies have been conducted (Bickman et al. 2011, 2014), resulting in a relatively narrow research base for the implementation of MFSs in children and young persons. Even in adult populations, in which MFSs have been studied more extensively (e.g. Davidson et al. 2014; Krägeloh et al. 2015; Shimokawa et al. 2010), we do not know much about potential unhelpful effects of feedback. A recent study suggested that providing feedback in a long-term inpatient and day patient psychotherapy setting, was initially associated with an increase symptoms in patients with borderline personality disorder and patients with personality disorder not otherwise specified, although these effects diminished after several weeks (De Jong et al., submitted). In youth mental health care, there may also exist groups of patients for which feedback is not helpful (e.g. developmental disorders, high severity clients). Additionally, new research suggests that feedback may also be unhelpful for therapists who are motivated by preventing failure (prevention focus), rather than obtaining success (promotion focus; De Jong and De Goede 2015). More research would be needed in order to study potentially unwanted effects of MFSs.

A different class of potential unwanted effects has to do with forming partnerships with the industry. Modern MFSs have become so technologically complex and costly that researchers often need to form partnerships with software developers in order to be able to develop a MFS (as for example was done by Bruns et al. 2015). Often, software developers are co-investors in these projects. Sometimes scientists buy into a MFS financially as well, or get benefits from the sales of a package, or from giving trainings in the use of the MFS. These situations may lead to conflicts of interest, or situations in which shared creative ownership of the MFS can lead to problems in the continuation of a research line (as Bickman et al. mention in their article).

## Conclusion

The articles in this special section teach us that the implementation of MFSs is a complex process in which many challenges need to be faced. They also show us good examples of successful implementation processes or creative solutions to challenges faced, which will be extremely helpful to future implementers of MFSs. The implementation of MFSs in routine care has the potential to improve outcomes for large groups of future clients. This is especially important, given that the effect sizes of interventions in routine care are much smaller than those found in clinical trials (Hansen et al. 2002; Weisz et al. 1995). Moreover, 21 % of children and young people deteriorate significantly during their care episode (Warren et al. 2009). This means that the result we are getting in routine care are not good enough. The implementation of MFSs in routine care is an important step in improving outcomes, that has the potential to affect quality of life for thousands of children and young persons worldwide. It should be mentioned that so far, the research on the use of MFSs in youth mental health care has primarily taken place in the US. More research in other countries is necessary, especially given the fact that care systems and accessibility of care is quite different in other countries. For instance, in many European countries, youth mental health care is either freely accessible, or for a relatively affordable co-pay. Additionally, the level of training for clinicians may also differ substantially between countries, which may impact both outcomes and the implementation process for MFSs. It is encouraging to see that implementation of MFSs in the US has taken flight, and hopefully the articles in this special section will inspire others to start thinking about implementing a MFS in their own setting.
